# Maternal depression, adverse childhood experiences, and social support in relation to gestational diabetes risk: results from the Albany Infant and Mother Study (AIMS)

**DOI:** 10.1186/s12884-021-03814-5

**Published:** 2021-04-27

**Authors:** Margaret Versteegen, Christine T. Bozlak, Heather Larkin, Allison A. Appleton

**Affiliations:** 1 University at Albany School of Public Health, Rensselaer, USA; 2 Health Policy, Management, and Behavior, University at Albany School of Public Health, Rensselaer, USA; 3 University at Albany School of Social Welfare, Albany, USA; 4 Epidemiology and Biostatistics, University at Albany School of Public Health, Rensselaer, USA

**Keywords:** Gestational diabetes, Psychosocial factors, Depression, Adverse childhood experiences, Social support

## Abstract

**Background:**

Psychosocial factors are of increasing interest as potential influencers in disease development. This study explores associations between gestational diabetes mellitus (GDM) and maternal depression, adverse childhood experiences (ACEs), and social support, in response to emerging evidence in these areas.

**Methods:**

An observational, prospective cohort study (AIMS) served as the source of secondary data for this study. Participants included 300 pregnant women aged 18–40 years at an upstate New York prenatal care clinic, who completed a set of self-report questionnaires assessing exposures and stressors both during and prior to their pregnancy. Data were also abstracted from infant and maternal medical records.

**Results:**

Logistic regression modeling estimated the odds ratios (ORs) of developing GDM in relation to psychosocial factors. There was a significant association between depression and GDM (OR = 2.85, 95% CI: 1.15, 7.06), which persisted in the model adjusted for age and BMI (aOR = 3.19, 95% CI: 1.25, 8.10). No significant associations were found between ACEs or social support with GDM.

**Conclusions:**

Study findings support an association between maternal depression and GDM development. This study underscores the need for additional research on psychosocial factors and connections to health risks.

## Background

Gestational diabetes mellitus (GDM) is a form of diabetes, or abnormal blood glucose (sugar) metabolism, diagnosed during pregnancy. Pregnant, non-diabetic women who are initially noted to experience high blood glucose levels during their pregnancy are considered to have GDM [1]. The causes of GDM are complex and not fully understood, yet it is becoming a common pregnancy complication with significant implications for infant and mother morbidity and mortality, in addition to developmental concerns later in life.

GDM prevalence rates have increased nationwide, affecting 9.2% of all United States’ (US) pregnancies [13]. Some common risk factors have been identified, primarily with regards to maternal demographics and clinical characteristics, like higher age and body mass indices (BMIs). However, a review of the literature reveals some discrepancies and variations in these risk factors, including a number of underexplored psychosocial risk factors.

The biopsychosocial model [15] acknowledges the importance of taking a more holistic view of individuals and health outcomes, and prominently takes into account factors that affect health from not just the biological sphere, but equally from the social and psychological domains. Limited research has been conducted on psychosocial factors, behaviors, and early life exposures and their possible latent effects on pregnancy. This provides an important area of focus for intervention measures, and a better understanding of risk relationships is necessary.

Psychosocial factors, like depression, adverse childhood experiences (ACEs), and social support, have been linked to a wide range of health outcomes. Maternal depression is a risk factor for several different negative pregnancy outcomes and has been linked to GDM in recent research [6, 7, 19, 20]. Depression has also been shown to have a negative association with health behaviors that might mitigate GDM risk in pregnant women, including healthy dietary intakes, exercise, and stress-management [26].

ACEs refer to stressful or traumatic events that occur before age 18, and ramifications of the cumulative stress from ACEs have been found to increase adult risk for several chronic diseases including coronary heart disease, stroke, obesity, and depression [8]. Negative experiences during critical childhood and adolescent developmental periods can create a life-long burden that may be particularly evident during pregnancy. Mason et al. [23] appear to have been the first to test for associations between GDM and two of the four ACE domains, physical and/or sexual abuse (categorically measured using the Revised Conflict Tactics Scales), finding a dose-response relationship with GDM risk that did not attenuate with adjustment for overweight/obesity.

Low well-being [29] and unhealthy nutritional intakes [26] have been linked with low levels of social support in pregnant women, and these can have a negative impact on pregnancy concerns like GDM. While pregnancy is a natural physiological process, it can be an overwhelming experience for some women, and is replete with physical and emotional changes. Pregnancy adaptation has been found to be enhanced in women with higher levels of social support, and these women reported significantly less stress compared to women with lower levels of social support [9]. Emerging evidence suggests connections between maternal social support and GDM risk. Mizuno et al. [24] found a woman’s level of emotional support and neighborhood trust was significantly and independently associated with GDM prevalence during the second or third trimester; this illuminates how poor emotional support can be stressful and cause hormone release, leading to increased insulin resistance in the body.

Research on connections between depression and GDM is still evolving, and limited with regards to associations with ACEs and social support. The objectives of this study were to further explore associations between depression and GDM, and address literature gaps considering ACEs and social support as contributors to GDM risk.

## Methods

Data were derived from the Albany Infant and Mother Study (AIMS), an observational, prospective cohort study. Between June 2015 and February 2018, 300 pregnant women who received prenatal care at an obstetrics clinic in upstate New York were enrolled on average at 27 weeks gestation. Details of this study can be found elsewhere [3]. Inclusion criteria were singleton pregnancy, maternal age between 18 and 40 years, English-speaking, and planned to give birth at the clinic’s medical center. All participants provided documented informed consent and completed a self-report questionnaire packet. After birth, a structured abstraction of clinical information regarding maternal health, delivery, and infant characteristics was conducted by physicians. Of the 300 who enrolled, 266 had available information on all psychosocial study variables and were included in analysis. Twenty-two women (8.3%) had a diagnosis of GDM. The study protocol was approved by the University at Albany Institutional Review Board.

The present analysis considers three different psychosocial predictor variables measured by validated instruments and includes: the Edinburgh Postnatal Depression Scale (EPDS [12];), the ACES questionnaire [16], and the Interpersonal Support Evaluation List (ISEL [10];).

The EPDS is comprised of ten questions that assess depressive symptoms in the past week, each scored on a four-point scale (0–3) for possible scores ranging from 0 to 30 [11]. The EPDS is widely used and has been validated in women during pregnancy (α = 0.82 [5];), with higher scores indicative of more depressive symptoms. Participant scores were considered on a continuum and as categorical predictors. Given that participants completed the EPDS primarily during their second trimester, depression was dichotomized as ‘yes’ and ‘no’ with presence of depression defined as a score > 10 [5].

The ACES questionnaire is a 10-item self-report tool developed after the original ACE study by Felliti et al. [16, 17]. It provides a retrospective identification of childhood trauma and stressful experiences that occurred prior to age 18, with total points scored out of ten. Given the extensive literature that has shown a variety of negative health outcomes with a graded, dose-response relationship with ACEs [8], participant ACE scores were used as a continuous measure. ACE scores were also dichotomized into high (≥4) and low (< 4), as a significantly increased risk for negative health outcomes has been identified in those with an ACE score ≥ 4 [17]. Analytical power was too low to provide stable estimates with the 5-category and abuse category ACE variables (data not shown).

The ISEL [10] is a widely used measure of general social support developed in response to the identified moderating effect of certain factors on stressful and/or negative life events, and the desire to better quantify the positive role of social support. Higher ISEL-12 survey scores indicate greater perceived social support. ISEL-12 scores typically range from 0 to 36; in the AIMS dataset, derived scores ranged from 12 to 48 and were used for analysis in this current study for ease and continuity. ISEL scores were examined continuously and categorically. No ‘cut-off’ values have been established for the ISEL-12, so tertiles of the sample scores were used to establish categorical groups with ‘low’ (bottom two tertiles) and ‘high’ (top tertile) social support.

The main outcome of GDM was diagnosed in study participants using a standard oral glucose tolerance test (OGTT); this was performed as part of routine prenatal care at the obstetrics clinic between 24 and 27 weeks, and results (yes/no) were extracted from medical records. Based on the literature, multiple covariates were considered as potential factors involved in GDM risk. Age and gestational weight gain were extracted from medical records. Participant BMI was calculated from the height and pre-pregnancy weight information documented in the medical records. Race, ethnicity, income level, education level, and marital status were obtained from the demographic survey portion of the study packet.

To test the hypothesis that maternal depression, ACEs, and social support are associated with GDM, sets of logistic regression models were fit for each construct with the binary GDM variable as the outcome. Odds ratios (ORs) and 95% confidence intervals (95% CIs) were estimated. For each psychosocial factor, unadjusted and models adjusted for covariates were fit. An unadjusted model was first created for the different predictor variables (Model 1), followed by a minimally controlled model using the age ≥ 30 covariate (Model 2), and finally a third model that controlled for both age ≥ 30 and BMI ≥ 30 (Model 3). All analyses were conducted using IBM SPSS Statistics, Version 25. A *p* value ≤0.05 was considered significant.

## Results

On average, participants were 28.5 years old, with a pre-pregnancy BMI of 29.02 (overweight category as per CDC guidelines). Examining BMI categorically, the two highest frequency categories were obese women (37.6%) and normal weight women (35.2%). Participants gained 12.01 kg (kg; or 26.46 pounds) on average during their pregnancy. Looking at pregnancy weight gain categorically, in terms of recommended weight gain based on pre-pregnancy BMI, the highest frequency category was those who gained more than the recommended amount of weight with almost half of all participants (47.5%). Just over half of study participants classified themselves as White, Non-Hispanic (52.4%), and 11.9% identified as being Latina/Hispanic. Many women were low-income; the most common annual income category reported by 19% of participants was <$9000. The majority of women had achieved a > high school education level (62.0%), and were not married (62.4%). Participant characteristics are presented in Table 1.

**Table 1:** Participant demographics

Continuous Variables	Mean	Standard Deviation
Age	28.54	5.48
Pre-pregnancy BMI	29.02	8.78
Pregnancy weight gain (kg)	12.01	7.26
Categorical Variables	Frequency	Percent of total
Race		
Black/AA, NH	75	25.5
White, NH	154	52.4
Other	65	22.1
Ethnicity		
Latino/Hispanic	35	11.9
Not Latino/Hispanic	259	88.1
Income level		
< $9000	56	19.0
$9000–19,999	38	12.9
$20,000–29,999	47	16.0
$30,000–39,999	19	6.5
$40,000–49,999	19	6.5
$50,000–79,999	22	7.5
$80,000–99,999	12	4.1
> $100,000+	39	13.3
Don’t know	30	10.2
Prefer not to answer	12	4.1
Education level		
≤ High School/GED	112	38.0
> High School	183	62.0
Marital status		
Married	111	37.6
Not married	184	62.4
Pregnancy weight gain (kg)		
Gained recm’d wt	72	27.8
Gained <recm’d wt	64	24.7
Gained >recm’d wt	123	47.5

*Abbreviations*: *kg* kilograms, *AA* African American, *NH* non-Hispanic, *GED* general education development test

Preliminary bivariate analyses (data not shown) indicated a significantly higher risk for GDM in women aged ≥30, and in women with obesity (pre-pregnancy BMI ≥ 30); these covariates were thus used in regression models described below. No significant associations were seen in bivariate analyses for race, ethnicity, education, income, marital status, categorical pregnancy weight gain, or when categorizing age or BMI in additional ways, and thus were not added to the regression models testing the associations between psychosocial factors and GDM.

The average EPDS score among participants was 8.70 (standard deviation (SD) 5.56). Examining binary EPDS scores, women with depression were found to be at significantly increased risk for GDM across all three models (Model 1, OR = 2.85, 95% CI: 1.15, 7.06, *p* = 0.023; Model 2, aOR = 3.10, 95% CI: 1.23, 7.77, *p* = 0.016; Model 3, aOR = 3.19, 95% CI: 1.25, 8.10, *p* = 0.015). The strongest association was found in Model 3, with an almost 3.2 elevated odds of GDM after adjusting for both age and pre-pregnancy BMI. No significant associations were observed when depression was treated continuously (Table 2).

**Table 2:** Logistic regression models for the association between depression and GDM

	Model 1		Model 2		Model 3	
	OR (95% CI)	R^2^	aOR (95% CI)	R^2^	aOR (95% CI)	R^2^
EPDS total	1.05 (0.98, 1.14)	1.4%	1.05 (0.98, 1.13)	4.7%	1.04 (0.97, 1.13)	8.5%
EPDS binary	2.85 (1.15, 7.06)*	4.6%	3.10 (1.23, 7.77)*	8.5%	3.19 (1.25, 8.10)*	12.7%

Model 1: unadjusted.

Model 2: adjusted for age ± 30.

Model 3: adjusted for age ± 30, BMI ± 30.

*Abbreviations*: *OR* odds ratio, *aOR* adjusted odds ratio, *R*^*2*^ Nagelkerke R^2^

*: denotes finding is statistically significant, *p* ≤ 0.05

The average ACE score among participants was 2.72 (SD 2.576). Categorically, 67 women (26.3%) reported 0 ACEs, 44 (17.3%) reported 1 ACE, 37 (14.5%) reported 2 ACEs, 30 (11.8%) reported 3 ACEs, and 77 (30.2%) reported 4 or more ACEs. No significant associations were found in regression analyses for the various considerations of the ACE score variable and GDM (Table 3). Some trends towards increased GDM risk with the binary ACE variable were noted, although results were firmly nonsignificant.

**Table 3:** Logistic regression models for the association between ACEs and GDM

	Model 1		Model 2		Model 3	
	OR (95% CI)	R^2^	aOR (95% CI)	R^2^	aOR (95% CI)	R^2^
ACEs total	1.06 (0.90, 1.26)	0.4%	1.04 (0.88, 1.23)	4.2%	1.00 (0.84, 1.18)	9.3%
ACEs binary	1.83 (0.74, 4.55)	1.5%	1.61 (0.64, 4.05)	5.0%	1.31 (0.50, 3.39)	9.5%

Model 1: unadjusted.

Model 2: adjusted for age ± 30.

Model 3: adjusted for age ± 30, BMI ± 30.

*Abbreviations*: *OR* odds ratio, *aOR* adjusted odds ratio, *R*^*2*^ Nagelkerke R^2^, *ref* reference category

*: denotes finding is statistically significant, *p* ≤ 0.05

The average ISEL score among AIMS participants was 38.63 (SD 7.33). No significant associations were observed for social support (either measured continuously or dichotomously) and GDM (Table 4), although a slight trend suggesting a protective effect of high levels of social support during pregnancy for GDM development was noted.

**Table 4:** Logistic regression models for the association between social support and GDM

	Model 1		Model 2		Model 3	
	OR (95% CI)	R^2^	aOR (95% CI)	R^2^	aOR (95% CI)	R^2^
ISEL total	0.98 (0.93, 1.04)	0.3%	0.98 (0.93, 1.04)	3.7%	0.99 (0.93, 1.05)	7.7%
ISEL binary	0.61 (0.23, 1.61)	0.9%	0.60 (0.22, 1.59)	4.4%	0.64 (0.24, 1.73)	8.3%

Model 1: unadjusted.

Model 2: adjusted for age ± 30.

Model 3: adjusted for age ± 30, BMI ± 30.

*Abbreviations*: *OR* odds ratio, *aOR* adjusted odds ratio, *R*^*2*^ Nagelkerke R^2^

*: denotes finding is statistically significant, *p* ≤ 0.05

## Discussion and conclusions

The main finding of this study was that participants with EPDS scores indicative of depression were at higher risk for GDM. Preliminary bivariate analyses support the evidence base with regards to the connections between older maternal age and higher pre-pregnancy BMI with greater GDM risk. The association between depression and GDM risk was further strengthened after additional modeling adjustments for maternal age and BMI. While less robust than the depression findings, this work also suggests ACEs and social support during pregnancy may influence GDM risk. We encourage future research in this area.

Based on the extensive literature review conducted, this study appears to be the first prospective examination of the impact of multiple psychosocial factors, including maternal depression, ACEs, and social support, on the development of GDM. Research on health issues is increasingly acknowledging the contribution that psychosocial factors may have on the development of diseases, and it is critical to expand understanding in these areas to better inform preventative interventions.

While continuous EPDS scores did not show significant relationships with GDM, an examination of binary EPDS scores indicative of depression did reveal significant associations across all three models (Table 2). The fully adjusted Model 3 showed women with depression had an almost 3.2-fold increased risk of developing GDM, corroborating research in this area that also controlled for covariates of maternal BMI and age [6, 7]. In contrast, some literature notes a significantly higher risk for GDM in depressed women who were non-obese [19], or found that the relationship between GDM and depression became nonsignificant after controlling for weight-related variables like pre-pregnancy BMI and gestational weight gain [25]. These variations may be due to other population nuances.

Research has shown a likely bidirectional relationship between depression and diabetes, with both physiologic and psychosocial pathways [7], which is consistent with the biopsychosocial perspective. Metabolic control of diabetes has been shown to be affected by psychophysiological processes, as well as individual adherence to prescribed treatment regimens, inclusive of both medications and behaviors like diet and physical activity [28]. Stress levels and coping abilities influence metabolic control and these pathways [28]. Depression has been linked to hyperglycemia [22]; both hyperglycemia and insulin resistance are associated with GDM. Future research can better elucidate the relationship dynamics.

Given that by definition ACEs occur prior to age 18, these instances of adversity may be thought of as being remote to pregnancy, particularly regarding a study sample with an average age of 28.5 years. However, as our knowledge increases of the potential latent biological and psychosocial ramifications of ACEs [2, 8, 17], their possible influence via behavioral and/or physiological pathways related to pregnancy health warrants examination. While none of the models reached statistical significance, the binary ACE variable may hint at trends in support of this hypothesis (Table 3).

A connection between social support and GDM could also potentially occur directly through physiological processes, or indirectly through behavioral and/or psychosocial factors that impact physiology. There is evidence supporting the hypothesis that higher levels of maternal social support can decrease the risk for GDM through increased physical activity [18, 26, 30] and/or healthy lifestyle behaviors [21, 26]. However, the current study analyses examining associations between social support and GDM are firmly non-significant (Table 4), perhaps suggesting that maternal social support is not as relevant to the pathophysiology of GDM.

Pearson’s correlation assessments showed EPDS scores were highly positively correlated with ACE scores (significant at the 0.01 level, 2-tailed), and were highly negatively correlated with ISEL scores (significant at the 0.01 level, 2-tailed; data not shown). As a participant’s score on the EPDS increased, so did their total number of ACE categories, and their tendency to have a lower level of social support. This reinforces the strong interrelationship between these psychosocial variables, and suggests the potential for indirect effects from adverse events over the life course. This study found significant relationships between depression and GDM, but despite the strong correlations between maternal depression, ACE scores, and ISEL scores, significant relationships between ACEs or social support with GDM were not found in regression analyses. Recent explorations highlight that irrespective of duration or intensity, adversity exposures of both moderate and high levels during any period of childhood heighten the risk for depression later in life [31]. Bădescu et al. [4] report on the connection between increased chronic stress levels contributing to inflammatory pathways that cause insulin resistance. Women with low levels of perceived social support report more depressive symptoms, but research indicates that networks of strong social support provide a protective effect against pregnancy complications [14]. While this study finds an association between depression and risk for GDM, a history of ACEs or perceived level of social support may be a step removed from this risk relationship.

### Study limitations

This study has several limitations to note. The prevalence of GDM was low in the study population, which placed limits on analytical power and the ability to identify associations. A non-random, convenience sample comprised the study participants, and the OGTT used to diagnose GDM was performed as part of routine prenatal care, and was not specifically conducted as part of the study protocol. Additionally, measures of the psychosocial variables were obtained via self-report on the prenatal questionnaires. It is possible that different measurements of these variables would be obtained using clinical diagnosis. The generalizability of results may be limited given that all study participants were English-speaking and were receiving prenatal care.

### Study strengths & implications

Data for this study were collected prospectively, and included a multimodal data collection protocol which included both self-report questionnaires and medical record abstractions. Additionally, a large proportion of participants were low income and of minority race. Further investigation of psychosocial factors in larger, diverse populations is warranted, including examination of the impact of interventions at various time-points throughout the life course.

This study provides important contributions, specifically addressing some of the shortcomings in the knowledge base of the potential implications for psychosocial factors in GDM development. Findings support the literature connecting GDM and maternal depression, and that maternal age and BMI strengthens this association. These findings underscore the importance of considering both psychosocial factors and biological variables in GDM development. Given the small sample size, forthcoming work in larger, more diverse populations can replicate, test, and build upon these hypotheses.

Pantell et al. [27] examined the value of combining both clinical and social factors in order to more precisely predict risk; the results of regression models showed a graded association: as the number of social and behavioral risk factors increased, so did the risk of developing diabetes. Future research in women’s health should build upon these ideas to increase our understanding of the complex interactions between biological, psychological, and social health factors. Potential mechanisms for associations between the factors examined in this study could be further explored to determine causal or mediating/ moderating relationships. Additionally, interventions for psychosocial factors found to be associated with the development of GDM may show a greater impact on development and/or control of GDM as compared to current recommendations that focus primarily and/or exclusively on health behaviors like physical activity and diet. Taking a holistic, biopsychosocial approach may prove effective in guiding future research and interventions to address pregnancy complications like GDM.

## Data Availability

The datasets analyzed during the current study are available from the corresponding author on reasonable request.

## Abbreviations

ACEs: Adverse childhood experiences
BMI: Body mass index
EPDS: Edinburgh Postnatal Depression Scale
GDM: Gestational diabetes mellitus
ISEL: Interpersonal Support Evaluation List
OGTT: Oral glucose tolerance test
